# A Narrative Review of the Effects of Internal Jugular Vein Compression on Brain Structure and Function During Periods of Head Impact

**DOI:** 10.7759/cureus.77625

**Published:** 2025-01-18

**Authors:** Kathryn A Brieck, Zachary J Brieck, John A Ashby, Owen C Phelps, Ibolja Cernak

**Affiliations:** 1 Neurology, Augusta University Medical College of Georgia, Augusta, USA; 2 Neurology, Mercer University School of Medicine, Macon, USA; 3 Physiology, Thomas F. Frist, Jr. College of Medicine, Nashville, USA

**Keywords:** brain concussion, brain slosh, contact sport, head trauma, jugular vein, narrative review, protective equipment, sport related concussion

## Abstract

Subconcussive impacts are very common in the sports world and can have many negative impacts on human function, including increased risk for cognitive decline and behavioral impairments such as chronic traumatic encephalopathy (CTE). The purpose of this article is to analyze the available literature on the effects of jugular vein compression applied by a cervical collar on cerebral structure and function in the setting of chronic impact exposure. This narrative review analyzed 17 articles on brain structure and function, published between 1992 and 2022.

Our review of the 17 studies shows an overall neuroprotective effect of the external jugular vein compression applied by the cervical collar during insult to the head as compared to groups who did not wear a collar. These findings suggest a potential role of the cervical collar, in addition to helmets, in reducing the incidence of concussion-induced microtraumas and cascading secondary injury mechanisms.

Though positive results are consistent throughout the studies, future studies with increased sample sizes are necessary to create precise estimates of the effects of the cervical collar. In addition, the analyzed studies mainly looked at the effects of the cervical collar on football players, soccer players, and Special Weapons and Tactics (SWAT) team members; thus, additional rigorous studies are needed to assess the impact of the cervical collar on other high-risk populations such as military and law-enforcement personnel, among others.

## Introduction and background

Definition of concussion

A concussion, a common form of traumatic brain injury (TBI) [1], is defined by the Berlin definition of concussion as a “TBI induced by biomechanical forces transmitted to the head, often via a direct blow to the head, face, neck, or other part of the body.” In general, concussions cause a rapid onset of transient impairments of neurological function that will often resolve spontaneously. Acute clinical signs and symptoms reflect primarily functional rather than structural damage, though neuropathologic changes can occur. Symptoms and clinical signs may vary in presentation, and loss of consciousness is not required for diagnosis. Finally, the resolution of clinical and cognitive impairment is gradual, and some symptoms may be prolonged or even permanent [2,3]. Common physical symptoms of concussions include headache, balance issues, nausea, vomiting, tingling in the extremities, and seizure activity. Non-physical symptoms include memory impairment or “foggy thinking,” slowed thinking and reaction time, sleep disturbances, and mood swings [4].

Definition of subconcussion

A subconcussive impact (SCI), similar to a concussion, is caused by a traumatic biomechanical force delivered to the head or other parts of the body and transmitted to the head, but not reaching the necessary threshold to elicit concussive symptoms [5]. Consequently, a SCI does not cause clinical manifestations of concussion, nor does it lead to detectable brain damage, thus making the diagnosis and management challenging [6]. However, each SCI causes a degree of microtrauma, and the cumulative effect of repetitive traumas is of concern. Commonly noted long-term symptoms of SCIs include cognitive decline, emotional instability, and depressive symptoms [7]. 

Pathobiology of concussion and subconcussion 

Both primary and secondary mechanisms of injury are involved in concussive and subconcussive damage. The primary injury mechanism refers to the immediate mechanical forces, such as tissue stress and strain in the brain, directly following impacts. The secondary injury mechanisms follow the primary injury mechanisms and have a complex timeline and interwoven interactions potentially leading to neuronal death [8]. Activation of glutamate and subsequent excitotoxicity, inflammatory mediators, and modulators leading to a state of chronic inflammation; breakdown products of spectrin; endoplasmic reticular stress; mitochondrial dysfunction; and oxidative stress, among other mechanisms, have been seen to play important roles in secondary injury mechanisms [8-11].

Importance of concussions and subconcussions in sport

Both concussive and subconcussive impacts are common in contact sports, including American football, soccer, ice hockey, boxing, wrestling, rugby, and even baseball [1,12,13]. Though a single concussion does not usually cause permanent damage to the brain, multiple concussions can cause significant structural and functional changes [14,15]. Depression and depressive symptoms have often been reported in athletes with a history of sports-related concussions [15,16]. Other known effects include cognitive decline and various behavioral impairments as well as an increased risk of chronic traumatic encephalopathy (CTE). Furthermore, accumulating studies showed macrostructural, microstructural, and neurochemical changes in athletes. Interestingly, a study surveying former collegiate and professional athletes showed that the majority of them believed their functionality was not different from that of the general population. Nevertheless, a study assessing the neurological health of American football players suggested an increased risk of neurodegenerative diseases such as Parkinson’s, Alzheimer’s, and amyotrophic lateral sclerosis (ALS) [15]. 

Public interest in SCI-induced brain microtraumas began in 2002, when the autopsy of Jeffrey Astle, a prominent soccer player, showed extensive degenerative brain disease and tauopathy consistent with CTE, despite having no history of concussions. Although, historically, heading the ball was not considered injurious, his death was ruled as “death by industrial disease” from a repeated head injury. This has led to further research focusing on TBI/CTE in various sports [17,18]. It is now believed that, similar to concussions, an accumulation of SCIs over a career or throughout a lifetime can potentially lead to significant brain damage. It is noteworthy that patients with a history of SCIs can present similarly to those who suffer from repeated concussions, with the long-term effect of CTE or other neurodegenerative diseases such as Alzheimer’s disease and Alzheimer's disease-related dementias (AD/ADRD) [19] or others.

Though the proportion of players with TBI developing CTE is unknown [20], CTE is a serious health concern as it is a progressive neurodegenerative disease characterized by cognitive, behavioral, and motor abnormalities [21]. Mez and colleagues [22] showed evidence of CTE in 110 out of 111 autopsies of professional football players, demonstrating varying degrees of brain atrophy, astrogliosis, myelinated axonopathy, microvascular injury, perivascular inflammation, and phosphorylated tau protein pathology. These findings raise significant health concerns for athletes and other professions potentially exposed to SCIs.

The bilayered jugular vein compression (JVC) collar is a new device designed as protective equipment for athletes. Its size is determined by measuring the circumference of the neck to ensure correct placement in relation to the internal jugular vein (IJV). Namely, when properly positioned, the collar acts to dilate the IJV above the collar, which, in turn, increases intracranial blood volume [23]. This “backflow” of blood acts to reduce the brain’s unrestrained movement in the skull (“slosh”) [24]. The reduction in “slosh” is due to the “cushioning” effect of the blood from the engorged IJV, decreasing brain motion in the skull, thus decreasing the energy transfer to the brain upon collision [24,25]. The purpose of this review is to condense the existing literature regarding the effects of the JVC collar on brain structure, including changes in white matter as well as histological markers of damage, and on brain function, measured by modalities such as electroencephalograms (EEGs) and working memory tasks.

This article was presented as a poster at the Georgia Academy of Family Physicians on November 10, 2023.

## Review

Data collection and analysis

Search Strategy

Articles for this narrative literature review were selected over the course of three main steps. First, a PubMed search was independently conducted using the search term "Jugular Vein Compression Collar." The initial search yielded a total of 26 articles with no filters applied, all of which were reviewed by the four researchers involved in data acquisition. Results from 26 articles were discussed between members. Inclusion criteria consisted of original articles that were quantitative in nature, written between the years of 1992-2022, and explicitly focused on the jugular vein compression collar’s impact on brain structure and/or function during periods of head impact.

Exclusion Criteria

**Figure 1:**
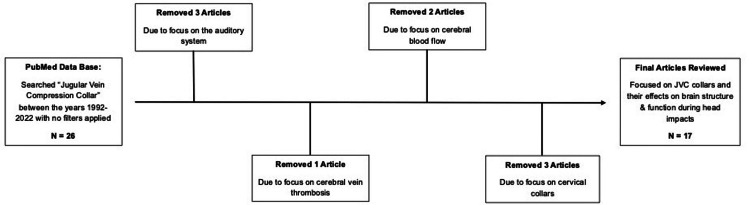
The general study design, illustrating the selection process of the original 17 articles. JVC: jugular vein compression

Exclusion criteria consisted of non-original studies, studies with little/no focus on brain structure or function, and studies that were qualitative in nature (Figure 1). Next, articles were analyzed and removed based on relevance to the desired topic of JVC collars and their effect on brain structure and function during the periods of head impact. This resulted in the removal of nine articles: three articles were removed due to their focus on the auditory system; one article due to focus placed on cerebral vein thrombosis rather than concussive and subconcussive impacts; two articles due to their focus on cerebral blood flow with no relation to concussive and subconcussive impacts; and finally, three articles due to their focus on cervical collars and not JVC collars. Consequently, the removal of these nine articles yielded a total of 17 articles that were specifically related to our topic of JVC collars and their effect on brain structure and function following periods of head impact.​ 

Results

Brain Composition 

White matter: Multiple studies investigated white matter (WM) alterations in the athletes’ brain composition following periods of head impact. Diffusion tensor imaging (DTI) was used to compare WM alterations in subjects before and after their relative exposures to head impacts [19,23,26-29]. These studies showed that individuals not wearing a JVC collar had significant white matter changes following their respective sports season as compared to their brain’s structural features at the beginning of the season. Specifically, some studies found significant decreases in mean, axial, and radial diffusivity [19,23,26,27,29]. Other studies found an increase in mean diffusivity and/or axial diffusivity, a change from baseline nonetheless [28]. In comparison, in the vast majority of studies, individuals wearing a JVC collar did not experience any significant changes in their WM microstructure integrity [19,23,27-29]. It is noteworthy that WM changes partially resolved in the following months for those in the non-collar group, which demonstrated that the changes were mostly functional and not structural impairments. However, in multiple cases, these changes still remained significantly different from the baseline even after partial healing [19,27]. 

It is also worth noting that one study found longitudinal increases in fractional anisotropy and axial diffusivity in both the collar and non-collar groups. They also found that preventative effects were only noticeable once a minimum threshold of impact was met for the collar groups [26].

In addition to DTI, WM changes were also studied with neurite orientation and density imaging (NODDI) by Yuan et al. [29] as they measured WM before and after blast exposure in Special Weapons and Tactics (SWAT) personnel. NODDI is a novel MRI technique that acquires diffusion-weighted data from different areas in the microstructural components of the brain. Specifically, neurite density index (NDI), isotropic volume extraction (FISO), and orientation dispersion index (ODI) were calculated. While there was no significant difference between ODI and FISO scores between collar and non-collar groups, this study found a statistically significant difference in NDI scores. The NDI assesses the packing density of axons or dendrites, and the scores were found to be significantly higher in those without the collar [29]. Previous studies have indicated that an increase in NDI scores is associated with brain injury [30,31], with increased scores potentially reflecting neuron repair or changes in tissue water following neuronal injury [32,33].

Histological markers of injury: Experiments were done on rats and swine to measure the effects of JVC on histological markers of brain injury after head impacts [24,25,34]. Among them: Turner and colleagues [24] measured neural degeneration (Fluoro-Jade B), gliosis (glial fibrillary acidic protein), and neuroinflammation (ionized calcium-binding adapter molecule 1); Smith [25] measured amyloid precursor protein (APP, an indication of axonal injury); and Mannix [34] measured levels of the phosphorylated tau epitope AT8 (an indication of potential CTE) and the inflammatory microglial marker IBA1 (an indication of microglial activation) across the entire brain. The abovementioned three studies found statistically significant differences in histological markers of brain damage between groups with JVC and those without. Specifically, the JVC groups showed less expression of the respective histological markers as compared to the control groups without the compression, indicating that jugular compression provided some level of neuroprotection. 

Sindelar and colleagues [35] focused on the relation between JVC and intracranial hemorrhage. Using a group of swine subjected to controlled cortical impact, the neuropathologists graded the histological markers of brain damage, including beta-amyloid precursor protein levels, the persistence of degenerative/neurons, mediators of inflammation, and cerebral edema via hematoxylin and eosin (H&E) stain, as well as the presence and intensity of subarachnoid and intraparenchymal hemorrhages. The results showed that while there was no significance between collar and non-collar groups in the measurement of histological markers, there was a statistically significant decrease in the subarachnoid- and intraparenchymal-hemorrhage scores in the JVC group [35]. Thus, the authors concluded that mild internal jugular vein compression reduced subarachnoid and intraparenchymal hemorrhage in the porcine-controlled cortical impact model.  

Brain Function 

Electroencephalogram (EEG): In 2018 [36], Bonnette and colleagues performed a study to determine the ability of the JVC collar to mitigate blast-induced EEG changes. Given that axonal electrical signaling is affected by diffuse axonal injuries, they proposed that EEG would be able to indirectly measure axonal integrity by measuring changes in electrocortical signaling of the brain using a comparison of time spent in laminar or constant state vs. variable or flexible state. The variable or flexible state is indicative of unfocused, free thought, while the laminar, constant state is indicative of focused thought. A healthy brain has a consistent ratio of laminar to variable flow. This study showed that those without JVC had significant changes between pre- and post-blast EEGs, whereas the EEG patterns in those with JVC did not change after the blast. The non-JVC group showed longer periods of laminar electrocortical activity with intermittent bursts of variation rather than continuous variation. Put simply, the ratio of time spent in either state of EEG activity changed. The researchers hypothesized that this may have an effect on the brain’s ability to adapt to change or new environments, as the optimal variability argument is a theoretical framework for neuroplasticity. This study builds on previous findings published by Myer and colleagues [37], which showed stable and unchanged brain network activation (BNA) measured by EEG in JVC groups during the entire hockey season as compared to a significantly increased BNA score in the non-JVC groups. These studies reported a potential of the JVC collar to prevent statistically significant TBI-induced changes in brain network dynamics. 

Functional magnetic resonance imaging (fMRI) and working memory:* *In 2017, Yuan and colleagues [38] performed a study involving 52 high school football athletes and found that athletes without JVC showed significantly larger changes in BNA pre- vs. post-season as compared to athletes with JVC. They also reported that the non-JVC groups showed significantly stronger BNA in 13 brain regions during the N-Back tests in the post-season compared to pre-season, including the bilateral precuneus and superior parietal gyrus, supramarginal gyrus, precentral gyrus, superior frontal gyrus, middle frontal gyrus, inferior frontal gyrus - triangular, superior occipital gyrus, middle occipital gyrus, and angular gyrus. Results were most profound in three areas: the precuneus, responsible for memory, information integration, cue reactivity, and response to pain [39]; the inferior parietal cortex, responsible for spatial attention, multimodal sensory integration, and oculomotor control [40]; and the dorsolateral prefrontal cortex, responsible for executive control functions such as task switching, task-set reconfiguration, inhibition, planning, working memory, and prevention of interference [41]. 

Pre- to post-season differences in BNA were not significant in the JVC group, potentially suggesting that the changes in BNA could have been prevented by JVC use. In this particular study, the increased BNA among the non-JVC group correlated with the total number of head impacts experienced throughout the season. The JVC groups did not experience such a change. Furthermore, the non-JVC groups showed a significantly increased fMRI BOLD signal in affected areas, which also correlated with the number of impacts experienced throughout the season in nine of thirteen brain areas. No similar observations were made in the JVC groups. In terms of cognitive task performance related to accuracy and/or response time, there was no significant difference between JVC and non-JVC groups at any point, measured either pre- or post-season [38]. 

The same group replicated their 2016 research design in a follow-up study testing female soccer players, and the results were very similar. Although the level of head impacts was similar between the JVC and non-JVC groups, BNA measured during the N-Back working memory task was significantly increased in the non-JVC group compared to those with JVC. In the female athletes, unlike the male football players, there was no correlation between the total number of impacts and the difference in BNA. However, this study showed a significant negative correlation between the pre-to-post-season changes in the non-JVC group’s fMRI activation and task accuracy, with task accuracy decreasing with BNA increase. The response time and task accuracy measured in the JVC group remained unaffected [42]. Resting-state fMRI (rs-fMRI) was also shown to be increased in non-JVC female athletes following a single soccer season compared to those who wore the collar [43]. In another study conducted on adolescent male football players, Narad et al. found a very mild but significant protective effect of the collar on working memory as measured by scores on the altering network score (ANT). While no other working memory exams (digital trail-making test, cued task switching) found a significant protective effect for the collar vs. non-collar group, the data inconclusively suggested that the collar was mildly protective in these metrics as well [44]. 

Similar effects can again be seen in a study done with SWAT personnel who were subjected to low-level blasts [45]. After blast exposure, non-JVC groups showed significantly higher fMRI brain activation during the N-Back working memory task, whereas the JVC showed no change in activation. There was a significant positive correlation between the average amplitude of the peak impulse measured by a sensor placed in the helmet during the exposure and the degree of increased fMRI signal within the non-JVC group. No such finding was found in the JVC group, implying that there is no significant structural change. 

Though there is mixed data, previous studies have indicated an association between increased fMRI signals and brain injury [46-51]. Additionally, there is data suggesting that greater working-memory task-related activation (i.e., activation during N-Back tests) is present in post-TBI patients, potentially reflecting compensatory or residual activation to reach previous levels of functioning [52]. The lack of change in brain function and activation patterns seen in JVC groups suggests a protective role.

Discussion 

Brain Composition

White matter: By measuring the WM composition of the brain before and after lengthy periods of subconcussive impacts, researchers saw a statistically significant difference between groups with JVC collars as compared to those without. In terms of the NODDI study, Churchill and colleagues previously found increased levels of NDI, specifically in the corpus callosum and internal capsule, to be associated with TBI [30], which is congruent with findings of increased NDI in rodent models with TBI [31]. This indicates that the JVC collar may have had a protective effect on SWAT personnel, as they did not see an increased NDI, while their counterparts who did not wear the collar had increased NDI. In acute TBI, it is possible to see a decrease in NDI, which may reflect the immediate axonal degeneration from injury. However, an increased NDI, as discussed, may be suggestive of long-term change and potential recovery via remyelination or sustained changes in tissue water [32,33].

Previous literature has also indicated that a decrease in WM volume measured by DTI is causally related to TBI [53-58]. However, other studies don’t support this conclusion, arguing that increased signals measured via DTI have been found in patients with mild TBI (mTBI) or athletes with concussions/subconcussions [59-61].

These discrepancies could be due to biological differences such as age or sex. Additionally, the type of sport, head impact exposure, and subsequent injury severity might also play a role. Possibly, the most important factor underlying the differences in findings is the timing when the DTI images have been collected [62]. Still, it remains that some of the published data supports the potential neuroprotective effect of JVC by preventing the TBI-induced reduction in WM. It remains unclear if the collar has a minimum threshold of impact for which it could be beneficial.

Histological markers of brain injury: A broad variety of molecular, biochemical, and histological parameters have been confirmed as reliable indicators of brain injury. Measuring impaired phosphorylation of the tau protein as well as different forms of unfolded/misfolded proteins has been among the popular methods of establishing brain damage. Indeed, high concentrations of these and other markers have been found in people with Alzheimer's disease and related dementias (AD/ADRD), as well as TBI-induced neurodegeneration [63]. 

APP is an inflammatory protein associated with the chronic phase of TBI. Heavily present in patients with TBI, this protein is also found in several neurodegenerative diseases such as Parkinson's disease (PD) and AD/ADRD. It has been established that accumulation of APP prevents effective protein travel across cell membranes, impairing mitochondrial and cellular function [64]. In addition, a history of TBI has been linked to increased susceptibility to developing PD [65,66] and frontotemporal dementia [67]. Gardner and colleagues in their 2018 study [68] showed that veterans with a history of mTBI had a 56% increased chance of developing PD. The above-mentioned studies would imply that the use of a cervical collar eliciting JVC could potentially protect against the development of neurodegenerative diseases later in life. 

Microglial marker IBA is often used as a biomarker indicating the activation level of microglia in the brain [69]. Previous research has suggested the role of activated microglia in the pathogenesis of TBI-induced neuronal deficits [70]. In their 2020 study, Mannix and colleagues used a swine model of mTBI to investigate the effects of JVC on histopathological outcomes after injury. They showed that JVC reduced the concentration of microglial marker IBA1, suggestive of diminishing the TBI-induced neuroinflammation and, thus potential neuronal dysfunction after TBI [70]. 

Brain Function  

EEGs: EEGs, which measure brain impulses, are commonly used to assess healthy brain function. Both studies showed that groups not wearing a collar exhibited higher levels of EEG activity [36,37]. This is noteworthy because research indicates that increased EEG activity is indicative of brain injury [71,72]. Considering the higher EEG activity in non-collar groups compared to collar groups in these studies, there is a compelling reason to believe that wearing the collar can prevent these dangerous elevated levels of electrical activity in the brain as indicated by EEG measurements.

fMRI and working memory: Prior literature has demonstrated the ability of the fMRI imaging modality to detect subtle abnormal brain activation patterns associated with working memory. Numerous studies have shown increased fMRI activity after TBI, specifically in the frontal lobes and areas relevant to working memory [46-51]. However, the relation between increased brain activation and TBI, as seen in the studies discussed in this review, is not clear, since there are also studies that show hypoactivation in these same areas (i.e., the frontal cortex) in TBI patients [73,74]. It is possible that these discrepancies stem from differences in the length of time between the onset of TBI and when the study was performed. It is also possible that the differences are partially attributable to the degree of brain injury present in the study subjects. Presentations and cognitive effects might differ between mild/moderate and severe TBIs, and Sanchez only studied patients with severe TBIs. Finally, Sanchez included only patients with diffuse brain injuries, whereas other studies included patients with focal and diffuse lesions [73,74]. However, it is important to note that the distinction between these two types of injuries is somewhat artificial, as the two are not mutually exclusive and often coexist. A recent study including patients with moderate and severe TBI showed 50% of patients had both focal and diffuse damages [51], and neuronal cell death has been shown to be present regardless of the type of brain injury [51]. Additionally, a previous study comparing pre- and post-game fMRIs of collegiate rugby players [5] found a decrease in functional connectivity patterns in the brain after short-term exposure to subconcussive impacts. A history of a previous concussion was also noted to change the brain’s response to subsequent subconcussive impacts. In conclusion, evidence supports the notion that brain injury results in altered patterns of connectivity and information processing. 

In the available literature, there are conflicting data about the effects of TBI on working memory performance: while some studies report no significant difference between TBI and normal groups [38], others observed decreased working memory performance in participants with TBI [42,44]. Possible explanations for these differences include discrepancies in the time of testing related to the traumatic insult, as well as the type and severity of TBI. Moreover, it might also be possible that significant memory deficits develop later, perhaps years after this insult. Finally, it is unclear how long the athletes experience neurological deficits and compensate for them before they become aware of or accept them [46-48,51,73,74].

Of note, protective effects were seen in groups of males, females, and various age groups, ranging from adolescents playing sports to middle-aged SWAT personnel. Given the volume of prior research indicating that increased fMRI activation is associated with TBI, we believe wearing a JVC collar could prevent or diminish TBI-induced working memory impairments. 

Limitations

In the process of reviewing these research articles, we identified several relevant limitations. Several studies had a relatively small sample size, limiting the power of the findings, and thus negatively influencing their generalizability. Additionally, we noted that few studies included female subjects, whereas the majority of them used male subjects; this might limit the overall generalizability of the findings to a broader population. 

The FDA’s approval of the use of a JVC collar widely opened further research opportunities; for example, examining the longitudinal effects of the JVC collar and its role in mitigating CTE. Follow-up brain imaging studies could be done years after wearing the collar to further analyze its potential protective effects on brain structure and function. Furthermore, currently, it is unclear whether the collar’s effectiveness depends on the type of neural damage (e.g., focal vs. diffuse injury) or mechanisms of the physical force (impact vs. acceleration/deceleration, angular vs. linear acceleration, or blunt vs. blast, among others).

## Conclusions

Based on the collected data, we believe there is evidence supporting the use of a JVC collar to prevent structural and functional damage to the brain due to traumatic insult(s) to the head. The effects of tearing and shearing forces that occur after head impact appear to be significantly decreased with the use of the JVC collar. Its placement over the internal jugular vein causes a backflow of blood into the cranium, creating an increase in intracranial blood volume and essentially cushioning the brain. This “cushion” then decreases the intracranial motion of the brain, thus preventing/reducing primary injury mechanisms. The 17 original studies included in this review showed decreased white matter changes, decreased histological markers of brain injury, and decreased pre-vs. post-season EEG and fMRI changes in those who wore the collar compared to those who did not. The implications of this potentially prophylactic effect are a massive step toward brain protection in athletes and professionals who have a high risk of acquiring head insults. However, larger sample sizes and longer interval studies are needed to assess the long-term effectiveness of the JVC collar and confirm its place among other protective measures.
